# Predictive value of 3D ultrasound assessment of endometrial receptivity for PGD/PGS for transfer pregnancy outcome

**DOI:** 10.1186/s12884-023-05534-4

**Published:** 2023-03-29

**Authors:** Kaixuan Sun, Yinling Xiu, Yinghua Wang, Tingting Yu, Xiaoli Lu, Xiliang Wang, Yuexin Yu

**Affiliations:** Department of Reproductive Medicine, General Hospital of Northern Theater Command, No. 5 Guangrong Road, Heping Area, Shenyang, Liaoning 110016 People’s Republic of China

**Keywords:** PGD/PGS, Pregnancy outcome, Endometrium, Window of receptivity, 3D ultrasound

## Abstract

**Objective:**

To investigate the predictive value of three-dimensional ultrasound assessment of endometrial receptivity in PGD/PGS transplantation patients on pregnancy outcome.

**Methods:**

280 patients undergoing PGD/PGS transplantation were enrolled and divided into group A and group B according to the patients’ pregnancy outcomes. The general conditions, endometrial receptivity indexes of the two groups were compared. Multifactorial logistic regression analysis was used to determine the factors influencing pregnancy outcome in PGD/PGS transplant patients. ROC curves were plotted to analyze the predictive value of 3D ultrasound parameters on pregnancy outcome. The results of the study were validated with patients who underwent FET transplantation, and the patients in the validation group were treated with the same 3D ultrasound examination method and treatment plan as the observation group.

**Results:**

The differences in basic situations between two groups were not statistically significant (P > 0.05). The percentage of endometrial thickness, endometrial blood flow, and endometrial blood flow classification type II + II were higher in group A than in group B (P < 0.05). Multifactorial logistic regression analysis showed that endometrial thickness, endometrial blood flow and endometrial blood flow classification were influencing factors of pregnancy outcome in PGD/PGS patients. The sensitivity of predicting pregnancy outcome based on the results of transcatheter 3D ultrasound was 91.18%, the specificity was 82.35%, and the accuracy was 90.00%, which has a high predictive value.

**Conclusion:**

3D ultrasound can predict pregnancy outcome by assessing the endometrial receptivity of PGD/PGS transplantation, in which endometrial thickness and endometrial blood flow have a good predictive value.

## Backgroud

Assisted reproductive therapy (ART) has given many couples the opportunity to have their biological children. Live births ranged from 40.1% per cycle initiated in women younger than 35 years to 4.5% live births per cycle initiated in women older than 42 years in the United States [1]. Successful pregnancy is a complex process and the key is embryo implantation, which depends on embryo quality, endometrial receptivity and synchronization of the two developments [2]. Embryonic factors are estimated to account for one-third of implantation failures, but with the maturation of in vitro embryo culture techniques, embryo quality has been gradually optimized and embryos can also be screened by means of microscopic morphological observation and preimplantation embryo genetic diagnosis/screening(PGD/PGS), which has greatly reduced the number of poor pregnancy outcomes due to poor embryo quality.

Endometrial receptivity(ER) refers to the combined ability of the endometrium to allow embryo positioning, adhesion, invasion implantation, growth, differentiation, metaphase and immune regulation during the window of implantation (WOI) approximately day 20–24 of menstruation, influenced by various regulatory factors [3–6]. To better understand and describe endometrial receptivity, Noyes et al. [7] first presented histological assays in the 1950s. In later years other endometrial assays including biomarkers, hormone receptors, and immunochemical markers gradually replaced Noyes’ criteria [8–14]. With the development of technology, the endometrial receptivity sequence was developed in 2011. Several modalities have been used to assess endometrial receptivity, with ultrasound being the most commonly used to assess endometrial morphology due to its non-invasive nature and availability [15].

Several previous studies have shown the predictive value of 3D ultrasound for in vitro fertilization-embryo transfer(IVF-ET) success, but those predictive value for embryo transfer success has been inconsistently stated [16–19]. These studies did not explore the relationship between endometrial tolerance and embryo quality. The aim of our study was to exclude the effect of embryo quality on pregnancy outcome, to investigate the predictive value of three-dimensional ultrasound assessment of endometrial tolerance in PGD/PGS transfer patients on pregnancy outcome.

## Materials and methods

### Study design and patients

Patients who underwent PGD/PGS transplantation for fertility treatment at the Department of Reproductive Medicine, General Hospital of Northern Theater Command from September 2016 to February 2020 were selected for the study. This was a retrospective study of routinely collected clinical data, and exemption from informed consent was approved by the medical ethics committee of General Hospital of Northern Theater Command (registration number: 202H2020PJ010). All experimental protocols were approved by the medical ethics committee of General Hospital of Northern Theater Command and all methods were carried out in accordance with the Declarations of Helsinki.

Inclusion criteria: (1) Receiving PGD/PGS transplantation for conception; (2) Regular menstrual cycle; (3) No infectious disease in the last 3 months.

Exclusion criteria: (1) History of previous uterine and ovarian surgery; (2) Combined with severe liver and kidney function diseases; (3) Combined with endocrine disorders such as hyperthyroidism and polycystic ovary syndrome; (4) Combined with endometriosis, adenomyosis, and uterine malformation of the uterus.

**Fig. 1:**
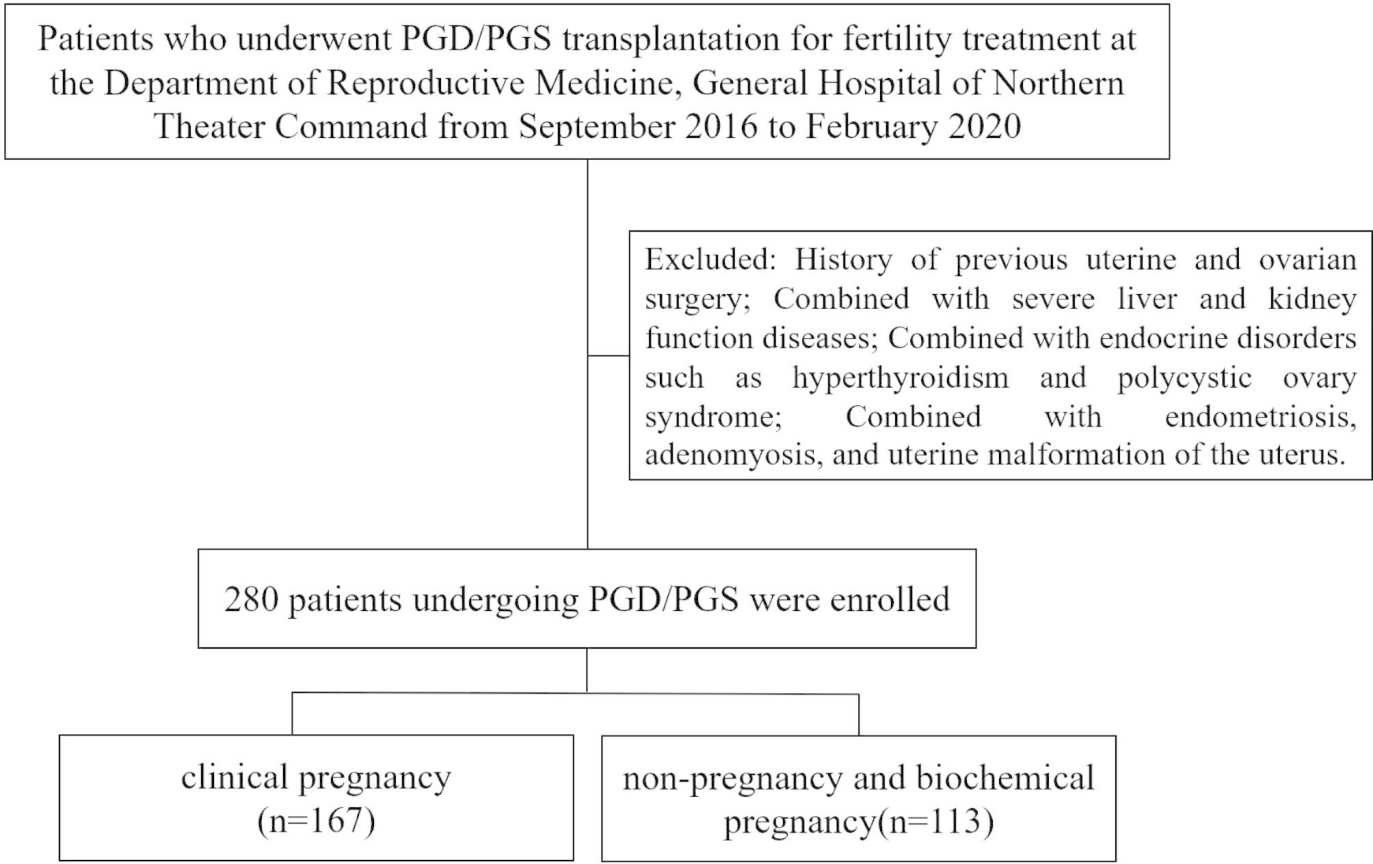
Flow chart of patient inclusion

All patients received individualized superovulation protocols. Patients were divided into group A (clinical pregnancy, n = 167) and group B (non-pregnancy and biochemical pregnancy, n = 113) according to their pregnancy outcome. Age of group A ranged from 24 to 42 years, mean (33.74 ± 4.12), years of infertility ranged from 0.1 to 20 years, mean 4.65 ± 3.88 years; 91 cases of primary infertility and 76 cases of secondary infertility. Age of group B ranged from 26 to 44 years, mean (34.04 ± 4.42), years of infertility 0.1 to 22 years, mean 4.69 ± 4.28 years; 53 cases of primary infertility and 60 cases of secondary infertility. There was no significant difference in the general data between the two groups (P > 0.05), and comparability existed (Fig. 1).

### Cycle protocol

Frozen-thaw embryo transfer(FET) protocols in the study center are mainly divided into natural cycle after spontaneous ovulation and artificial cycles (hormone replacement treatment cycles) based on the regularity of the menstrual cycle.

Endometrial preparation in the natural cycle group: Ultrasound was used on days 3–5 of the menstrual cycle to examine and exclude ovarian cysts, ultrasound was used on days 10–12 of the menstrual cycle to monitor follicle size, and when follicle diameter > 14 mm and endometrial thickness > 6 mm, blood estradiol (E2), follicle stimulation hormone(FSH), luteinizing hormone (LH) and progesterone (P) levels were monitored, and if necessary, intramuscular injection of chorionic gonadotropin (HCG) 10,000 U to induce ovulation and transfer blastocysts on the 5th day after ovulation. After ovulation, progesterone injection 40 mg/day was given intramuscularly and luteal support was provided until 11 weeks.

Endometrial preparation in the artificial cycle group: Endometrial thickness was monitored by ultrasound on days 3–5 of the menstrual cycle, and estradiol valerate 2 mg was given twice daily on the same day. This dose is adjusted based on endometrial thickness every 4 days. If the number of days of treatment is > 12 days and the thickness of the endometrium is > 6 mm, we will continue to give estradiol valerate tablets when the blood estrogen (E2) level is 200 pmol/L, and at the same time, we will inject progesterone 40 mg/day intramuscularly and transfer blastocysts after 5 days of endometrial transformation. Luteal support was continued after the completion of transfer. Embryos were resuscitated at the time of FET following vitrification and thawing procedures.

### Pregnancy

Non-pregnancy was HCG < 3mIU/ml 14 days after transplantation. Biochemical pregnancy was HCG > 20mIU/ml on day 14 of transplantation. Clinical pregnancy was observed by color doppler ultrasonography on the 21st day after transplantation. All clinical pregnancies were followed up to delivery.

### Ultrasound measurement

The frequency of transvaginal ultrasound probe was 7.5 MHz. On the morning of embryo transfer, the patient was asked to perform a vaginal 3D ultrasound scan after emptying the bladder. The examination included: (1) Endometrial thickness and morphology: The endometrial thickness was measured by taking the standard longitudinal image of the endometrium to the fundus of the uterus at 1.5 ~ 2.0 cm, and the average value was taken for three times, and the endometrium was classified into three types according to the morphology of the displayed endometrium: type A was the trilinear type, which showed a hyperechoic lateral line formed between the endometrium and the myometrium and two layers of the endometrium in close proximity to the surface. Type B is a transitional type, showing isolated echogenicity in the middle and inconspicuous midline echogenicity in the uterine cavity; Type C is a homogeneous strong echogenicity type, showing no midline echogenicity in the uterine cavity. (2) Uterine artery flow parameters: The ultrasound probe was placed on both sides of the cervix, and its spectrum was measured by pulsed Doppler at the most obvious point of colored blood flow, and the endometrial vascularization index (VI), blood flow index (FI), and vascularized blood flow index (VFI) were obtained by the software that comes with the machine. (3) Subendometrial blood flow branch typing and parameters: The standard endometrial longitudinal images were selected, endometrial blood flow was observed by energy Doppler, the number of intraendometrial perforating vessels was counted, the parameters of subendometrial spiral artery blood flow were measured by energy Doppler ultrasound, and the resistance index (RI), pulsatility index (PI), and the ratio of peak systolic velocity to end-diastolic velocity (S/D) of subendometrial blood flow were recorded. Endometrial blood flow was typed using the Applebaum typing method [20]. Type I: no blood perfusion was seen in both the endometrium and subendometrium; Type II: blood perfusion was seen in the subendometrium, but the blood flow did not reach the midline; Type III: blood perfusion was seen in the subendometrium and reached the midline. The direction of endometrial peristaltic movement was classified as (1) stationary; (2) non-stationary (①positive movement from the cervix to the fundus; ②negative movement from the fundus to the cervix; ③phasic movement; ④irregular movement).

### PGD/PGS assay

aCGH technology or NGS technology was used. Whole genome amplification was performed using the PicoPLEX Whole Genome Ampification kit (Rubicon, USA). aCGH was performed using Agilent G4900DA scanner for microarray scanning and BlueFuse Multi software for data analysis. NGS was sequenced using a high-throughput sequencer NextSeq CN500 (Berry Hutchinson, China). Embryos with deletion and/or duplication > 10 Kb were considered as aneuploid embryos and were discarded; embryos with amplification failure, no detection signal after loading or those that could not be judged according to the test results were considered as failed quality control embryos, and the decision to biopsy them again for PGD/PGS testing was made in communication with both patients; embryos without deletion and/or duplication were considered as euploid embryos and were judged as usable embryos, which were thawed and transferred at a later date.

### Statistical methods

All data were analyzed using SPSS 22.0 to compare the data of patients in group A and group B. The measurement data were expressed as mean ± standard deviation and t-test was used to compare the differences in ultrasound characteristics such as endometrial thickness, number of endometrial blood flow branches, endometrial peristaltic frequency and various blood flow parameters between the two groups. Count data were compared using chi-square test and Fisher’s exact probability method to compare the differences in ultrasound characteristics such as endometrial morphology, endometrial blood flow classification, and peristaltic direction. The area under the curve (AUC) was calculated using the subject operating characteristic curve (ROC) to analyze the value of endometrial characteristics and blood flow parameters in predicting pregnancy outcome in patients with FET. AUC > 0.9 was considered highly accurate, and 0.7 ≤ AUC ≤ 0.9 was considered more accurate, and a prediction model was established using logistic regression. *p* < 0.05 was considered a statistically significant difference.

## Results

### General information

The 280 PGD/PGS patients were divided into group A (167 clinical pregnancies) and group B (113 non-pregnant and biochemical pregnancies) based on pregnancy outcome. The differences in age, body mass index, years of infertility, reasons for PGD/PGS-assisted pregnancy, and type of infertility between the two groups were not statistically significant. The basal FSH was 7.25 ± 3.97 mIU/ml in group A lower than 8.51 ± 5.40 mIU/ml in group B (*p* < 0.05) (Table 1).

**Table 1:** Basic characteristics of patients in groups A and B. BMI, body mass index; PGD/PGS, Preimplantation embryo genetic diagnosis/screening; FET, frozen embryo transfer

Characteristics	A	B	*p*
years	33.74 ± 4.12	34.04 ± 4.42	0.552
BMI(kg/ml^2^)	23.59 ± 3.78	23.75 ± 3.30	0.724
Baseline FSH(IU/L)	7.25 ± 3.97	8.51 ± 5.40	0.026
Duration of infertility(years)	4.65 ± 3.88	4.69 ± 4.28	0.941
Type of infertility			0.213
Primary infertility	91(54.5%)	53(46.9%)	
Secondary inferitility	76(45.5%)	60(53.1%)	
Diagnoses of PGD/PGS			0.223
1.Chromosomal translocations, inversions and numerical abnormalities	12(7.19%)	5(4.42%)	
2.Recurrent miscarriages	58(34.73%)	33(19.76%)	
3.Repeated implant failures	36(21.56%)	20(17.67%)	
4.Advanced age	61(36.52%)	55(48.67%)	
FET protocols			0.343
Artificial cycle	147(88.00%)	95(84.07%)	
Natural cycle	20(12.00%)	18(15.90%)	

### Endometrial tolerability under 3D ultrasound

The endometrial thickness in the two groups was 0.90 ± 0.06 mm in group A, which was higher than 0.78 ± 0.13 mm in group B(*p* < 0.05). The number of endometrial blood flow was 8.35 ± 1.86 in group A higher than 6.82 ± 1.59 in group B(*p* < 0.05). The endometrial blood flow classification was 12 cases (7.18%) of type I endometrium and 155 cases (92.82%) of type II + III endometrium in group A and 18 cases (15.93%) of type I endometrium and 95 cases (84.07%) of type II + III endometrium in group B(*p* < 0.05). Endometrial morphology was 48 cases (28.7%) for type A endometrium and 119 cases (71.3%) for type B endometrium in group A patients, and 26 cases (23.0%) for type A endometrium and 87 cases (77.0%) for type B endometrium in group B patients(*p* > 0.05). The hemodynamic parameters VI, FI, VFI, uterine artery flow parameters RI, PI, S/D, and endometrial motion were not statistically significant in group A compared with group B (Table 2).

**Table 2:** 3D ultrasound endometrial receptivity parameters of patients in group A and group B

Characteristics	A	B	*p*
Endometrial thickness(mm)	0.90 ± 0.06	0.78 ± 0.13	0.000
Endometrial pattern			0.286
Type A	48(28.7%)	26(23.0%)	
Type B	119(71.3%)	87(77.0%)	
VI	5.99 ± 4.06	6.17 ± 4.19	0.748
FI	31.39 ± 6.90	34.20 ± 21.90	0.168
VFI	2.18 ± 1.61	2.25 ± 1.54	0.744
RI	0.81 ± 0.05	0.82 ± 0.05	0.224
PI	2.08 ± 0.43	2.12 ± 0.42	0.497
S/D	5.64 ± 1.53	5.88 ± 1.65	0.238
Endometrial blood flow	8.35 ± 1.86	6.82 ± 1.59	0.015
Endometrial blood flow classification			0.020
I	12(7.18%)	18(15.93%)	
II + III	155(92.82%)	95(84.07%)	
Endometrial Movement			0.517
Stationary	103	74	
Non-stationary	64	39	

VI Vascularization index, FI Flow index, VFI vascularized blood flow index, RI Resistive index, PI Pulsatility index, S/D the ratio of peak systolic velocity to end-diastolic velocity

### Factors influencing pregnancy outcome in PGD/PGS patients

Multi-factor logistic regression analysis with statistically significant variables in univariate comparisons as independent variables and pregnancy outcome as dependent variable showed that endometrial thickness (OR = 4.815, 95% CI: 1.381 to 16.788), number of endometrial blood flow branches (OR = 1.204, 95% CI: 1.008 to 1.438) and endometrial blood flow classification (OR = 2.122, 95% CI: 1.036 to 4.347) were influential factors for pregnancy outcome in PGD/PGS patients (Table 3) OR: odds ratio.

**Table 3:** Multifactorial Logistic Regression Analysis of Pregnancy Outcomes in PGD/PGS Patients. B: beta; S.E: standard error; Sig: significance test. OR: odds ratio

Characteristics	B	S.E	Wals	Sig.	OR	95%CI
Baseline FSH	-0.46	0.029	2.580	0.108	0.955	0.902 ~ 1.010
Endometrial thickness	1.572	0.637	6.085	0.014	4.815	1.381 ~ 16.788
Endometrial blood flow	0.186	0.091	4.187	0.041	1.204	1.008 ~ 1.438
Endometrial blood flow classification	0.753	0.366	4.233	0.040	2.122	1.036 ~ 4.347

### Predictive value of vaginal 3D ultrasound for pregnancy outcome

**Fig. 2:**
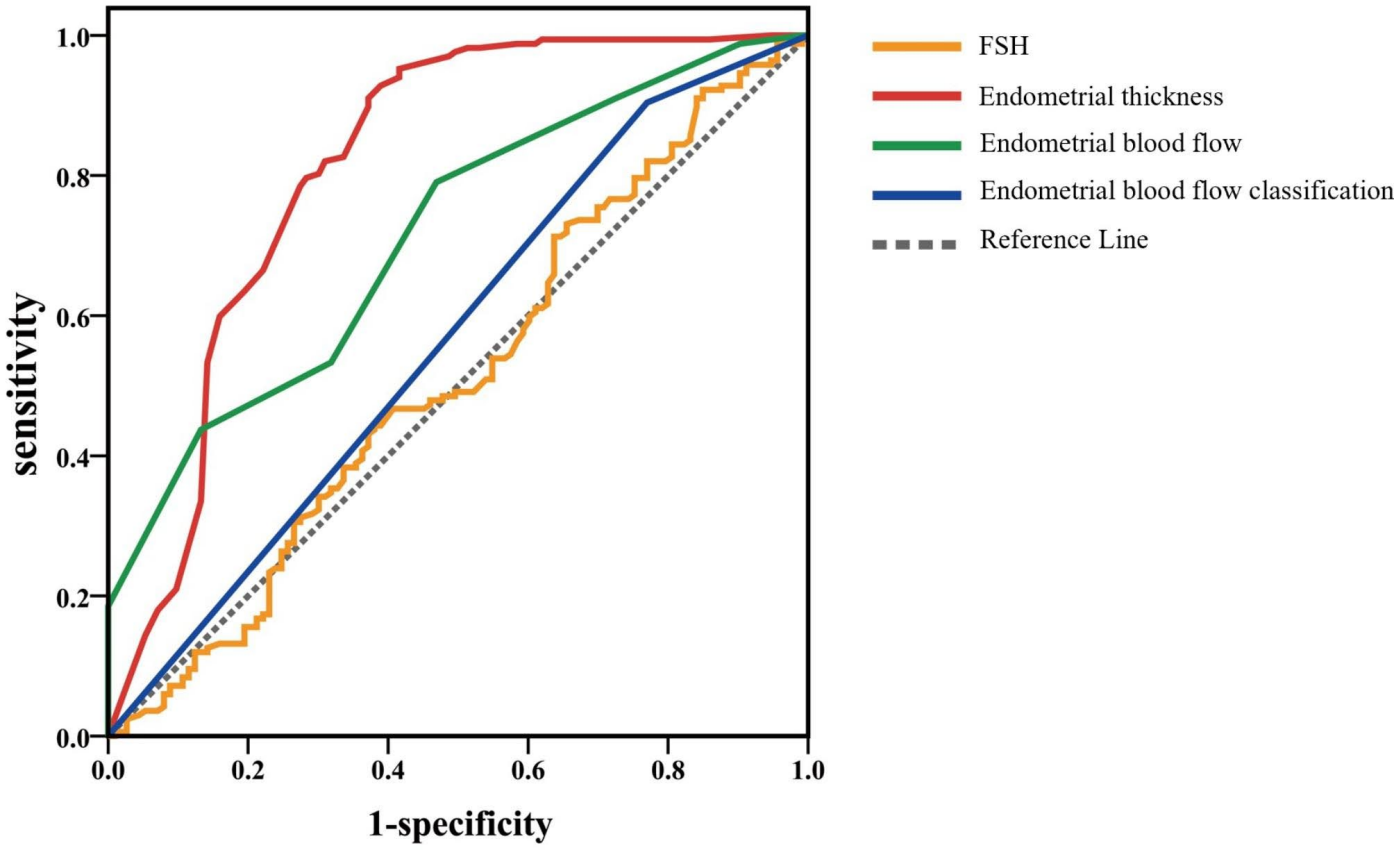
ROC curves for parametric prediction of pregnancy outcome. The ROC curve was plotted with pregnancy outcome = 1 and no pregnancy = 0. The results showed that EMT and endometrial blood flow branch number had predictive value for pregnancy outcome in PGD/PGS transplant patients. FSH Follicle Stimulation Hormone

The ROC curve was plotted with pregnancy outcome = 1 and no pregnancy = 0. The results showed that endometrial thickness and endometrial blood flow had predictive value for pregnancy outcome in PGD/PGS transplant patients. The area under the curve for endometrial thickness was 0.812 (95% CI: 0.756–0.869), and when the cut-off value was 0.88, the sensitivity and specificity were 0.802 and 0.720, respectively. The area under the curve for endometrial blood flow branching to predict pregnancy outcome was 0.714 (95% CI: 0.714), and the sensitivity and specificity were 0.790 and 0.531, respectively, when the cut-off value was 7.5. The area under the curve for FSH and endometrial blood flow classification to predict pregnancy outcome was 0.513 (95% CI: 00.443–0.583) and 0.567 (95% CI: 0.498–0.637), with no predictive value for clinical purposes (Fig. 2).

### Validation analysis

The results of transvaginal 3D ultrasound prediction against the actual human outcome are shown in Table 3. 68 clinical pregnancies and 32 non-pregnancies and biochemical pregnancies were eventually achieved in the 100 patients in the validation group after treatment. The endometrial thickness cut-off value of 0.88 and the endometrial blood flow cut-off value of 7.5 were used as references to predict 66 clinical pregnancies and 34 non-pregnancies and biochemical pregnancies. The sensitivity of predicting pregnancy outcome based on the results of transcatheter 3D ultrasound was 91.18% (62/68), the specificity was 82.35% (28/34), and the accuracy was 90.00% (90/100), which has a high predictive value (Table 4).

**Table 4:** Comparison of predicted outcomes of 3D ultrasound with actual pregnancy outcomes of patients in the validation group

Actual pregnancy outcome	Number of cases	Predicted pregnancy outcome		Total
		Clinical pregnancy	Non-pregnancy and biochemical pregnancy	
Clinical pregnancy	68	62(62%)	6(6%)	68%
Non-pregnancy and biochemical pregnancy	32	4(4%)	28(28%)	32%
Total	100	66(66%)	34(34%)	90%

## Discussion

Current methods of ER assessment based on morphology, molecular biology, proteomics, and gene chips are generally invasive, time-consuming, and require assessment one menstrual cycle in advance, which does not allow for timely pregnancy guidance for transplantation cycles. Noninvasive methods and indicators for endometrial receptivity assessment have become a pressing need in assisted reproductive medicine. Ultrasound assessment has the advantage of being clinically noninvasive, easy and timely, and highly operable, and is considered to have a potential role in the assessment of ER [15]. With the development of ultrasound technology, especially the use of 3D ultrasound and Doppler has provided a simple, noninvasive and reproducible method for clinical assessment of endometrial tolerability. More and more ultrasound indices are being used to visualize the endometrium and to try to find indices with predictive value for ER, and many different studies have emerged. Previous studies have mostly focused on the effect of ER on pregnancy outcome for IVF-ET or IUI and have not excluded the effect of embryo on pregnancy outcome. The present study focuses on the assessment of ER before transfer in patients undergoing PGD/PGS transfer, excluding the effect of embryonic factors on pregnancy outcome and focusing on the effect of ER on pregnancy outcome. Our study is more focused and innovative compared to other studies.

Endometrial thickness (EMT) is one of the commonly used indicators for ultrasound evaluation of endometrial receptivity, but its predictive value for embryo transfer success has been inconsistently stated in previous studies. Some authors suggest that clinical pregnancy rates in IVF-ET are positively correlated with EMT, and that as endometrial thickness increases, patients have higher pregnancy and implantation rates, lower spontaneous abortion rates, and higher live birth rates. Numerous studies have shown that an excessively thin uterus does not facilitate embryo implantation, leading to difficulties in conception. Patients with an EMT ≤ 7 mm or < 8 mm on HCG trigger day have a decreased clinical pregnancy rate, a spontaneous abortion rate of more than 50%, and a significantly lower live birth rate [21, 22]. Some studies have also found no correlation between endometrial thickness and pregnancy rates. For example, Weissman et al. found a decrease in both pregnancy and implantation rates after the endometrium exceeded 14 mm [23, 24]. It has also been studied that there is no correlation between EMT and pregnancy rate [25], but suggests that the minimum value of EMT required for pregnancy is 6.9 mm [26]. It is controversial whether EMT alone can be used as a predictor of pregnancy outcome [27]. In our study it was concluded that EMT correlated with pregnancy, with patients in the pregnant group having higher EMT than in the non-pregnant group. The inclusion of EMT in the multifactorial regression analysis resulted in an OR = 4.815, 95% CI: 1.381 to 16.788, and the inclusion of ROC analysis showed an area under the curve of 0.812 (95% CI: 0.756 to 0.869), demonstrating that EMT can be used as an independent factor to predict pregnancy outcome in PGD/PGS transplantation cycles.

A good endometrial blood supply is necessary for embryo implantation, and ultrasound detection of endometrial blood flow can directly reflect the microenvironment of the embryo implantation site and has an important predictive value for endometrial receptivity. It has been suggested that endometrial blood flow is strongly associated with successful pregnancy during ovulation-assisted cycles [28], and the clinical pregnancy rate was significantly higher in the group with detectable blood flow in both the endometrium and subendometrium on HCG day than in the group with no detectable blood flow [29, 30]. In this study, we found that the pregnancy rate of patients gradually increased with the increase of endometrial blood flow. The number of endometrial blood flow was higher in the pregnant group than in the non-pregnant group, and after including this index in the multifactorial regression analysis and ROC analysis, the results showed that endometrial blood flow had some predictive value as an independent factor for PGD/PGS pregnancy outcome. The proportion of type II + III blood flow was higher in the pregnancy group than in the non-pregnancy group. The area under the curve was found to be 0.567 (95% CI: 0.498–0.637) when endometrial blood flow classification was included in ROC analysis, respectively, which can be used to predict clinical pregnancy outcome in PGD/PGS embryo transfer, but its use alone has low accuracy in predicting clinical pregnancy rate. However, the endometrial 3D ultrasound blood flow parameters VI, FI, VFI, PI, RI, and S/D were not statistically significant; these parameters probe the overall vascularity and density of blood flow in the endometrium, and based on the presence of endometrial disruption in the selected cases in patients with lesions such as uterine adhesions, uterine polyps, and submucosal myomas, where patients still had pregnancy, it can be speculated that embryo implantation may be influenced by the blood supply to the endometrium at the local implantation site. It can be assumed that embryo implantation may be more influenced by the blood supply to the endometrium at the local implantation site rather than the overall blood supply to the endometrium. Because this study is a pre-PGD/PGS transfer ultrasound endometrial receptivity data, compared to IVF/ICSI transfer there are higher requirements in terms of endometrial receptivity in terms of blood flow, the transfer was cancelled when endometrial blood flow classification did not reach type II or III, and endometrial blood flow was too low, and the endometrium was readjusted and then prepared for transfer again, resulting in some bias in the data. The effect of ultrasound flow parameters on pregnancy outcome in PGD/PGS transplantation needs to be further investigated by collecting more samples.

Pregnancy outcome in IVF-ET patients can be affected by a combination of multiple components and factors, including age, basal endocrine levels, etc. FSH is a gonadotropin, and insufficient FSH secretion during the follicular phase results in delayed development of the endometrium during the luteal phase, which is detrimental to embryo implantation. In this study, basal FSH results were lower in the pregnancy group than in the non-pregnancy group, and after considering this factor as an influential factor in pregnancy outcome in PGD/PGS patients, basal FSH was included in the multifactorial regression analysis and ROC analysis, and the results showed that basal FSH did not independently predict pregnancy outcome in PGD/PGS. In contrast to the results of previous studies, basal FSH was considered to affect ovarian reserve function, although to some extent, but mostly related to indirect effects [31]. One study found that the correlation between FSH/LH ratio and ovarian reserve function was more pronounced compared to FSH alone [32]. It has also been shown that basal FSH has little predictive value for ovarian responsiveness and continued pregnancy in IVF cycles, and is only significant in some patients with higher basal FSH (15 mIU/mL) [33].

In current studies using ultrasound for ER assessment, it is basically assumed that the timing of the graft window is fixed for different menstrual cycles of the patient, but this is not the case, and the timing of the graft window varies in different menstrual cycles. Therefore, finding a method of ER assessment based on individualization may be the direction of ER assessment studies. The study reports that an endometrial receptivity array (ERA) has been developed to predict ER and to personalize the optimal date for embryo transfer. The study is looking for a fast, accurate and less invasive clinical tool for personalized embryo transfer guidance [32, 34]. Hashimoto et al. [35]found the importance of using ERA to find individualized implantation windows by performing ERA testing in 50 patients with repeated transfer failures.

In conclusion, endometrial thickness, endometrial blood flow, endometrial blood flow classification, and basal FSH have different degrees of influence on pregnancy outcome in PGD/PGS patients, and endometrial thickness can be used as an independent factor to influence pregnancy outcome. In FET cycles, ultrasound may play a unique role in real-time localization of embryo implantation and endometrial blood flow detection, and combining the advantages of ultrasound, ERA, and modern molecular techniques will allow for more accurate assessment of ER and improved pregnancy rates in FET.

## Data Availability

The datasets used and/or analyzed during the current study are available from the corresponding author on reasonable request.
